# More holes, more contrast? Comparing an 18-gauge non-fenestrated catheter with a 22-gauge fenestrated catheter for cardiac CT

**DOI:** 10.1371/journal.pone.0234311

**Published:** 2020-06-08

**Authors:** Andreas Marco Fischer, Philipp Riffel, Thomas Henzler, U. Joseph Schoepf, Andres F. Abadia, Richard Robert Bayer, Holger Haubenreisser, Dante Giovagnoli, Alexander Kremer, Stefan O. Schoenberg, Joshua Gawlitza

**Affiliations:** 1 Institute of Clinical Radiology and Nuclear Medicine, University Medical Center Mannheim, Medical Faculty Mannheim, Heidelberg University, Heidelberg, Germany; 2 Division of Cardiovascular Imaging, Department of Radiology and Radiological Science, Medical University of South Carolina, Charleston, South Carolina, United States of America; 3 Conradia Germany, München, Germany; 4 Clinic of Diagnostic and Interventional Radiology, Saarland University Medical Center, Homburg, Germany; University of Bologna, ITALY

## Abstract

**Objective:**

To compare the performance of an 18-gauge nonfenestrated catheter (18-NFC) with a 22-gauge fenestrated catheter (22-FC) for cardiac CT angiography (CCTA) in patients with suspected coronary heart disease.

**Subjects and methods:**

74 consecutive patients imaged on a 2^nd^ generation dual-source CT with arterial phase CCTA were included in this retrospective investigation to either an 18-NFC or 22-FC. In comparison to the 18-NFC, the 22-FC has three additional perforations for contrast agent dispersal proximal to the tip. We examined the two groups for differences in their average attenuation in the right and left ventricles (RV, LV) and in the atrium (RA, LA) as well as in the proximal right coronary artery (RCA) and the left main coronary artery (LM). The averages were calculated for both the 18-NFC and 22-FC.

**Results:**

Catheters were successfully placed on the first attempt 97% (36/37) for 18-NFC and 95% (35/37) for the 22-FC. The following enhancement levels were measured: 22-FC (in Hounsfield-Units (HU)): RV = 203±29, LV = 523±36, RA = 198±29, LA = 519±38, RCA = 547±26, LM = 562±25; 18-NFC: RV = 146±26, LV = 464±32, RA = 141±24, LA = 438±35, RCA = 501±23, LM = 523±23; RV (p = 0,03), LV (p = 0.12), RA (p = 0.02), LA (p = 0.04), RCA (p = 0.3), LM (p = 0.33).

**Conclusion:**

No significant differences in attenuation levels as well as in image quality of the coronary arteries were found between NFC and FC. Nevertheless, the 22-gauge FC examinations showed significantly higher attenuation in the left and right atrium as well as the right ventricle. Patients with poor venous access may benefit from a smaller gauge catheter that can deliver sufficiently high flow rates for CCTA.

## Introduction

Coronary CT angiography (CCTA) is an application that has traditionally required relatively high contrast media injection flow-rates [1–4]. Thus, for any type of CT angiographic study, adequate peripheral intravenous access is necessary to ensure diagnostic, high-quality image acquisition. Patients undergoing CCTA typically have risk factors including smoking, diabetes, as well as the diseases associated with metabolic syndrome [5, 6]. Unfortunately, these factors not only have a damaging effect on the central vascular system, but also on the peripheral veins. In particular, the long-term influence of a diabetogenic metabolism with associated micro- as well as macroangiopathy is known for an increased risk of complications during the establishment of venous access [7–9]. Especially extravasation of these vulnerable vessels is a common complication when using catheter sizes conventionally utilized for high flow-rate contrast protocols [10]. While this may be an inconvenience in the setting of an elective routine examination, in the context of emergency examinations, this may be especially challenging. Depending on the protocol, an adult CCTA examination requires a contrast flow rate of 5ml/s or above with a typical catheter size of 18-gauge for adequate contrasting to best ensure safety and optimal image quality [11–13]. Recent studies have shown that fenestrated catheters (FC), which differ from traditional non-fenestrated catheters (NFC) in that they have additional, smaller diameter outlets just proximal to the terminal outlet of the catheter, allow greater contrast media flow by redistributing and therefore reducing the pressure occurring at the tip [14]. Nonetheless, currently there is no evidence, if these small FCs result in equivalent attenuation levels when compared to standard, 18-gauge NFC at high flow-rates in the context of CCTA.

Therefore, the aim of this study is to compare the performance of an 18-gauge NFC with a 22-gauge FC for CCTA in patients suspected coronary artery disease. Retrospective data evaluation will be used as part of an objective image quality analysis to determine whether sufficient contrast can be achieved with CCTA using smaller-lumen catheters. This is of particular clinical interest when elderly patients with multi comorbidities need a diagnostic CCTA, while this cohort often shows limited peripheral venous conditions that do not allow the application of conventional large-lumen catheters.

## Materials and methods

### Subjects

The study protocol of this retrospective investigation was approved by the local Institutional Review Board with a waiver of informed consent (approved through Mannheim Medical Faculty Ethics Committee 2. Approval Nr: 2013-818R-MA). The data was anonymized and retrospectively analyzed. Our study complied with the Declaration of Helsinki adopted by the World Medical Association (WMA) in 1964. A total of 74 patient CCTA examinations were analyzed in this study. The type of intravenous catheter used was identified via chart review along with data regarding catheter placement success and complications. Patients were included based on the following inclusion criteria: >18 years of age and CCTA performed between January 2016 and December 2017. Exclusion criteria were inadequate CCTA image quality, mainly caused by respiratory (n = 3) and cardiac motion artifacts artefacts (n = 2). These so-called "stair-steps-artefacts" can complicate sufficient density measurement at different locations and lead to incorrect measurement results.

### Intravenous catheter placement

**Fig 1:**
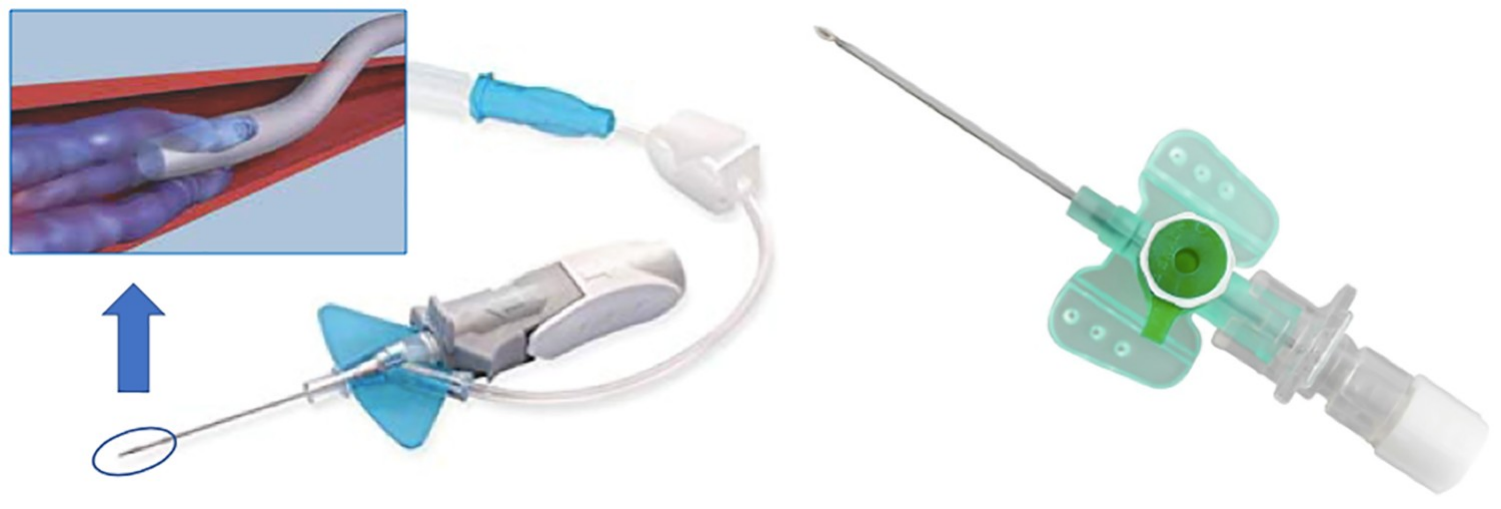
Comparison of both catheter systems. On the left side the catheter system with side holes is shown (22-gauge fenestrated). The figure above illustrates the flow technique after intravenous application as a model. On the righthand side a conventional catheter system (18-gauge non-fenestrated) with only one terminal hole is shown.

Two types of catheter were used: an 18-gauge NFC (18-gauge x 32 mm Venflon^™^ Pro Safety, Becton Dickinson) and a 22-gauge FC (22-gauge x 25 mm Nexiva^™^ Diffusics^™^ IV Catheter, Becton Dickinson). In comparison to the NFC, the FC has three additional outlets for contrast agent dispersal just proximal to the terminal outlet. For the 22-gauge, each of the additional outlets measures approximately 0.76 mm in length and 0.25 mm in width. Fig 1 shows a comparison of both catheter systems.

In our clinical routine, catheter placement is initially attempted in the antecubital region. In case of failed catheter placement, repeat attempts to each arm are made according to the patient’s tolerance. In cases where the catheter placement in the antecubital region was not successful, the examination was not included in the analysis.

### Coronary computed tomography angiography-protocol

Scans were performed in maximum inspiration on a 2nd generation dual source CT scanner (Definition Flash, Siemens Healthineers, Forchheim, Germany) at 120 kVp. The scan parameters were as follows: 120 kVp tube voltage, 50 mAs reference tube current using automated tube current modulation (effective mAs = 64 ± 31), 0.3 s rotation time, pitch 0.6, 128 x 0.6mm detector collimation. All images were reconstructed with a slice thickness of 1.5mm. Location of the catheter, CT-protocol, infusion rate, contrast volume and any catheter-related complications were recorded. Example complications include: exceeding maximum pressure, rupture of the perfusor extension line, contrast extravasation, or activation of the high-pressure alarm.

### Image quality assessment

**Fig 2:**
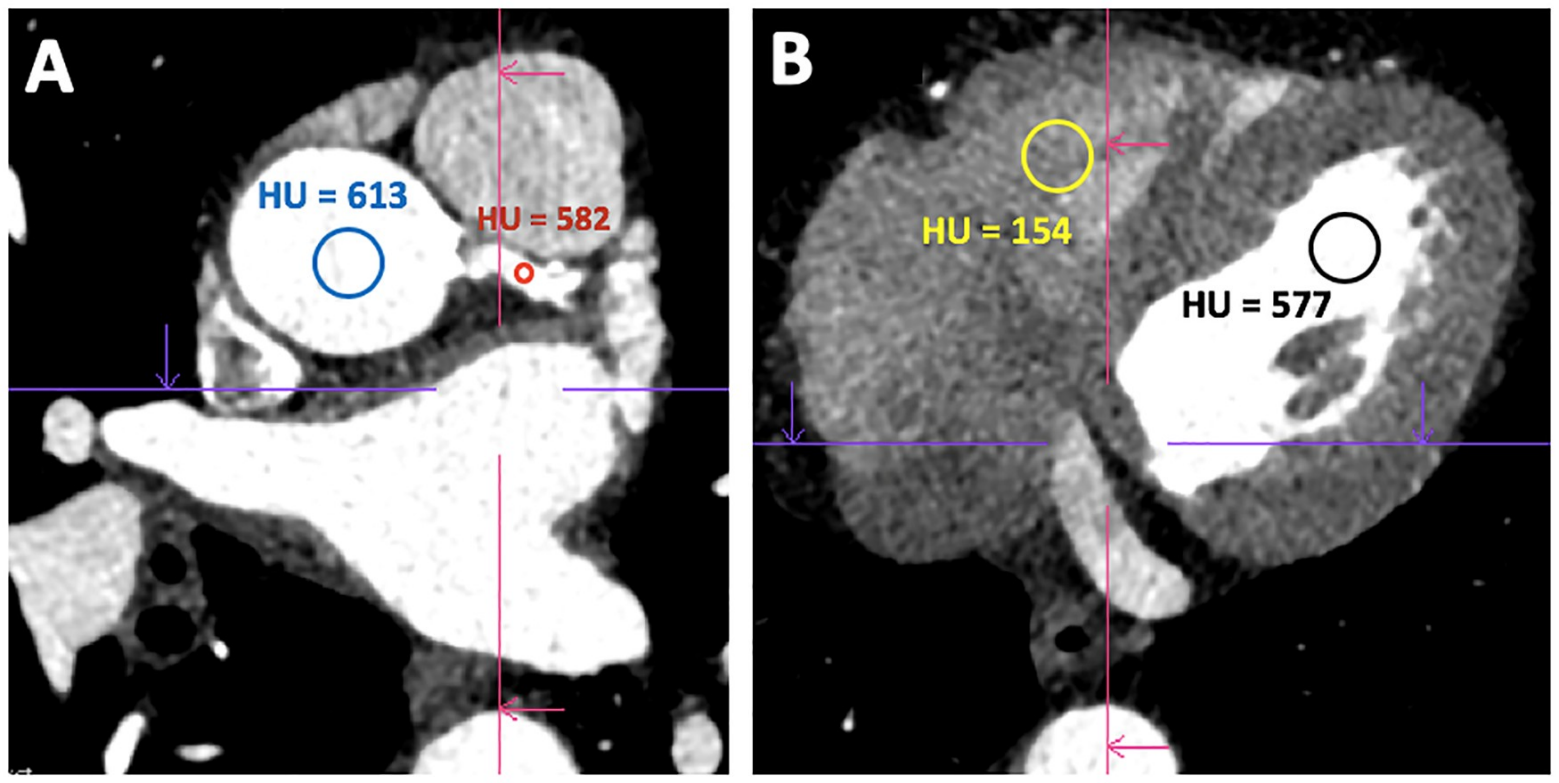
56-year-old male with a relevant cardiovascular risk profile (diabetes mellitus, arterial hypertension, hypertensive and diabetic nephropathy). Indication for CCTA due to intermittent chest pain; contrast medium application via 22-FC. Schematic presentation of the HU measurements taken via ROI in two different axial slices. Fig 2 A shows the HU measurement in the ascending aorta (blue) and in the LM (red). Fig 2 B shows the schematic HU measurement for the right (yellow) and left ventricle (black). CCTA = Coronary computed tomography angiography, FC = fenestrated catheter, HU = Hounsfield units, LM = left main coronary artery.

Images were evaluated for both objective and subjective quality by a radiologist (8 years experiences in cardiovascular imaging). The aortic enhancement levels were measured by a CT-technologist using regions of interest (ROI) and controlled by a resident physician (2 years experiences in cardiovascular imaging). A ROI which covered 50% of the lumen was placed in the diaphragmatic section of the aorta. Additionally, the enhancement levels of both atria and ventricles were measured in the CCTA. For this purpose, the ROI was placed in the respective anatomical region in the axial sectional image plane in order to subsequently record the HU values (Fig 2).

### Statistical analysis

All statistical analyses were performed using dedicated software (JMP 12, SAS, Cary, USA).

Averages were calculated for both the standard 18-gauge NFC and 22-gauge FC. Categorical variables were expressed using frequencies and percentages.

For both the 18-gauge NFC and the 22-gauge FC attenuation values were measured and the mean averages and standard deviations (SD) were calculated. A Student t-test was obtained to determine if there was a significant difference between the groups regarding their average attenuation.

Comparison of infusion rate, contrast volume and exceedance of maximum pressure was performed using individual two-sample Student t-tests as well. A p-value of less than 0.05 was considered statistically significant.

## Results

Patient examination of a total of 74 subjects (42 male) were included in this analysis. Between the 18-gauge NFC (n = 37) and the 22-gauge FC (n = 37) groups, there was no significant difference in age (p = 0.814), sex (p = 0.24), or contrast volume administered (p = 0.24) (Tables 1 and 2). The mean age of study participant was 55.9 ± 12.4 years (range: 18–81 years).

**Table 1:** Demographic summary.

Characteristics		18-Gauge Nonfenestrated	22-Gauge Fenestrated	p-value
Age (Years)	Mean	56.2	55.6	p = 0.814
	SD	11.2	13.6	
	Median	54	56	
	Min	34	18	
	Max	81	76	
Sex	Male	24 (65%)	18 (49%)	p = 0.24
	Female	13 (35%)	19 (51%)	
Risk factors	BMI > 30	8 (22%)	11 (30%)	p = 0.164
	CAD history	11 (30%)	13 (35%)	p = 0.625
	Smoking history	22 (59%)	25 (66%)	p = 0.476
	HLP	10 (27%)	11 (30%)	p = 0.800
	Hypertension	19 (51%)	23 (62%)	p = 0.355
	Diabetes	10 (27%)	8 (22%)	p = 0.594

BMI = Body Mass Index, CAD = Coronary artery disease, HLP = Hyperlipoproteinemia.

**Table 2:** Power injection summary.

	18-Gauge Nonfenestrated	22-Gauge Fenestrated	p-values
Flow rate (mL/s)	5.0	5.0	
Maximum pressure (325 psi) exceeded	1 (3%)	8 (22%)	p = 0.0281
Contrast agent delivered (mL)	80 ± 0	77.8 ± 2.2	p = 0.24

Contrast agent volume delivered is not significant; however, the difference in the rate of exceeding maximum pressure between the 18-gauge nonfenestrated and the 22-gauge fenestrated is significant.

The maximum pressure preselected with our contrast media injector (325 psi) was exceeded more often with the 22-gauge FC catheters (22%, 8/37)) than with the 18-gauge NFC (3%, 1/37)). This difference was statistically significant (p = 0.0281).

Of note, there was an additional unexpected complication documented that occurred in the examination protocols: the extension line used, ruptured three times during contrast administration with 22-gauge fenestrated catheters (8%, 3/37). When this occurred, a new catheter was used to obtain intravenous access and the examination was attempted a second time with the same parameters. In all cases, the second examination attempt was successful (100%, 3/3). This failure rate was not statistically significant (p = 0.24).

In all 74 CT-scans the subjective image quality was assessed and deemed acceptable. The aortic and cardiac enhancements levels could be measured for every subject (100%, 74/74).

**Fig 3:**
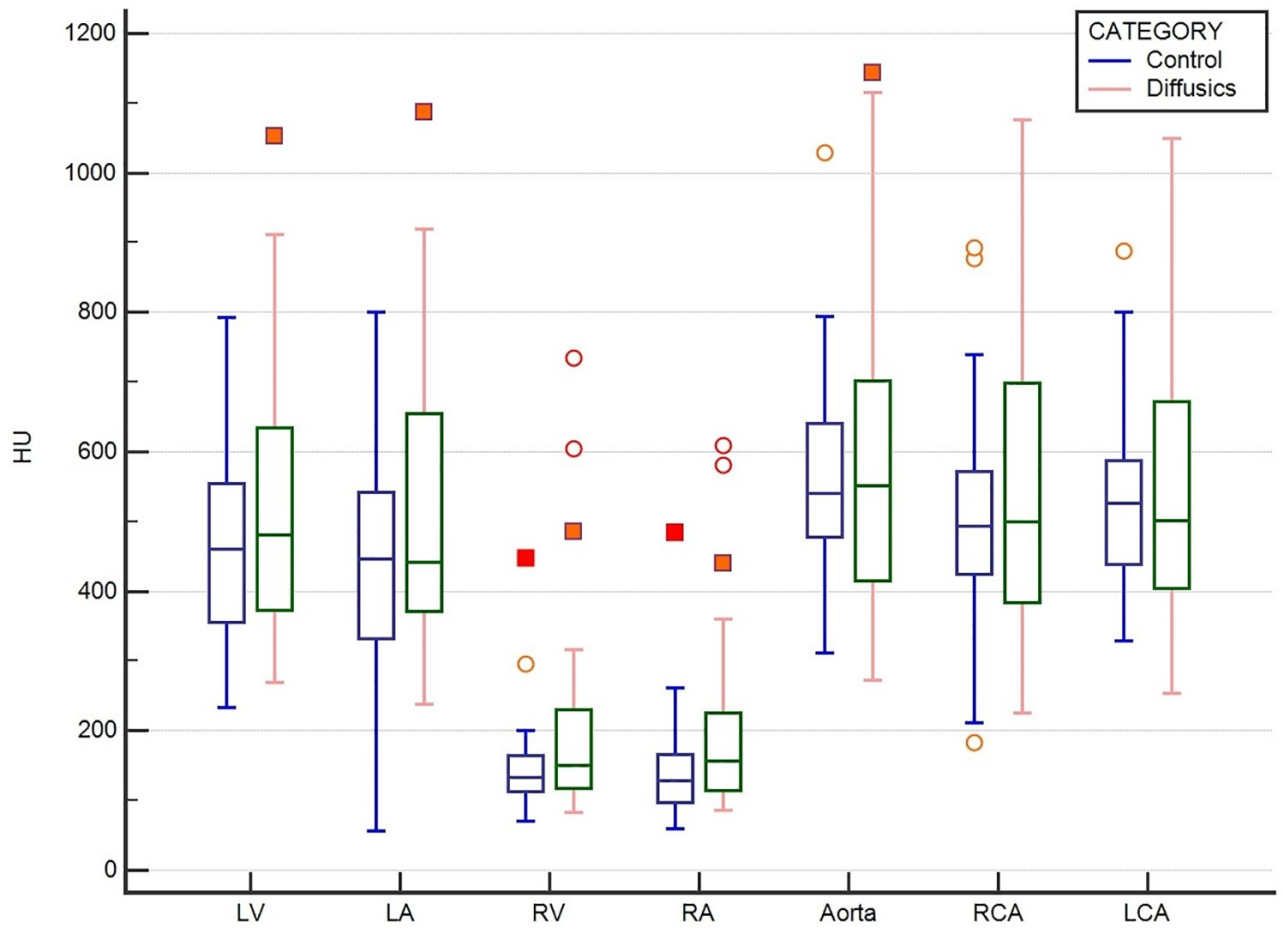
Box plots of different aortic enhancement levels from both catheter-types in Hounsfield Units (HU). Control = 18-gauge non-fenestrated catheter. Diffusics = 22-gauge fenestrated catheter. LV = left ventricle; LA = left atrium; RV = right ventricle; RA = right atrium; RCA = right coronary artery; LCA = left coronary artery.

Regarding the mean attenuation, significantly higher values for the FC were found in the ROIs of the left atrium (Ø 438 HU vs 519 HU; p = 0.04), right atrium (Ø 140 HU vs 198 HU; p = 0.02) and right ventricle (Ø 146 HU vs 202 HU; p = 0.03). No significant differences in attenuation were found for the ROIs of the left ventricle (p = 0.12), aorta (p = 0.28), right coronary artery (p = 0.3) as well as the left coronary artery (p = 0.3). All data of the comparison is shown in Table 3 and Fig 3 as well.

**Table 3:** Summary statistics of arterial phase aortic and cardiac enhancement levels (HU).

	18-Gauge Nonfenestrated	22-Gauge Fenestrated	p-value
Aortic density	550.0 ± 24.8	597.0 ± 25.7	0.28
Left Atrial Density	438.0 ± 35.4	519.4 ± 38.6	0.04
Left Ventricular Density	463.5 ± 31.9	523.3 ± 35.9	0.12
Left Coronary Artery	522.5 ± 22.6	562.2 ± 25.2	0.33
Right Atrial Density	140.8 ± 23.7	198.0 ± 28.9	0.02
Right Ventricular Density	146.1 ± 25.8	202.8 ± 28.8	0.03
Right Coronary Artery	500.6 ± 22.5	547.4 ± 25.8	0.3

Shown are the mean HU values with standard deviation as well as the individual measurements the regions of interest regarding fenestrated and nonfenestrated catheters.

## Discussion

CCTA has become increasingly common in clinical practice as an essential diagnostic component [15–17]. Particularly with CT angiographies, increasingly faster acquisition times require adequate assurance of the contrast agent application in order to ensure suitable contrast concentration during the arterial phase. For this purpose, practical CT protocols with correspondingly fast infusion rates including a specified minimum intravenous catheter size are needed. However, especially in multimorbid patients with significant cardiovascular risk profile (hypertension, diabetes, prior cardiovascular events) and thus limited peripheral venous status, the placement of a suitable catheter in clinical practice can be challenging. Based on our results, we were able to show that a 22-gauge FC is not inferior to the intravenous 18-gauge NFC in terms of safety and contrast flow.

In addition, there were no extravasations in either group. Some evidence suggests, as Wienbeck et al points to, an increased rate of extravasation in small-lumen 22-gauge catheters in the context of increased contrast medium flow [11]. However, it has also been reported that the simultaneous presence of additional outlets reduces the risk of extravasation of contrast agent by spreading the prevailing maximum pressure and concomitant velocity prevailing at the catheter tip to the remaining outlets [18, 19]. This is also consistent with the results of Weber et al., who found a 9–30% reduction in the speed of a sidearm catheter using a phantom study, due to decreased shear wall tension when the catheter is in an oblique orientation to the vessel wall [14]. They examined a 16-gauge catheter with an 18-gauge catheter, which had a different number of side outlets (2, 4, 6 or 8 side outlets) and slots (2 or 4 slots each with 2.5 mm). Contrast agent velocities of 5 and 10 ml/s were generated to investigate the effect on the phantom. The authors found, that heavy wall load as well as the speed of the contrast medium at the tip area for catheters with side outlets decreased noticeably. The in vitro results of the previous study have been validated on adult subjects in our study. An extravasation did not occur in any of our 74 subjects in both groups after inspection and exclusion of venous splints. However, one of the major complications in this study was the frequent triggering of the pressure alarm by the contrast medium pump on the 22-gauge FC. This can be explained by the fact that the pressure limit was reached faster with FC than with NFC due to the smaller diameter at the same contrast medium flow rate. Nevertheless, the permissible maximum pressure for the catheter was not exceeded at any time.

Several studies have shown a reduced risk of venous tear and consecutive extravasation in the presence of additional side-outlets, in contrast to the classic single-distal-opening catheter system, where the additional outlets allow flow rate and pressure to be distributed over several openings [20, 21]. Daniel et al. also pointed to a more even pressure distribution in the presence of a higher number of side outlets, as this decreases the contrast agent pressure at the distal opening [22]. In the study, plastic hoses were used to model the flow of the end outlet and the side outlets of different catheter designs. A smaller catheter system that accommodates a reliable and safe contrast agent application with higher infusion rates could significantly expand the patient population and comfort. Such newly developed catheters were well suited for our CCTA-protocols.

Johnson et al. examined contrasting of the aorta with an 18-gauge NFC and 20-gauge FC at an average infusion rate of 5.74 ml/s for the 18-gauge NFC and 5.58 ml/s for the 20-gauge FC [23]. Both types of catheters provided similar contrast infusion rates with no increased risk of extravasation for the 20-gauge FC group (20-gauge FC: 230.5 ± 27.6 HU, 18-gauge NFC: 215.6 ± 32.8 HU). In addition, the FC were still available to those patients who were considered ineligible for placement of an 18-gauge catheter due to venous status. Although the abdominal aorta was examined in this study and different catheter sizes were used when compared to our study focusing on the coronary arteries, the results are similar. To our knowledge only one study explored the feasibility of NFCs in cardiac imaging before [24]. Kim et al. compared 20-gauge NFCs and 22-gauge FCs in the context of cardiac imaging. As the authors pointed out themselves, 20-gauge catheters are not commonly used in CCTA as the limited flow rate has a direct impact on intravascular enhancement. Consecutively, a clear superiority of FCs regarding intravascular attenuation was shown. Besides the smaller catheters as reference group, a flow rate of 4 ml/s was used, which directly alters intraarterial contrast [25]. Beyond their results, we were now able to show that FCs are equivalent, and partially superior, to the larger 18-gauge catheters, suggesting further feasibility in clinical praxis even at high flow rates.

Our study underlies various limitations. First, catheter types were compared between different patients, but not individually in every patient. This is mainly due to the fact, that most patients did not receive multiple CCTAs at our medical center. Ideally, every patient would have received two scans: once with an NFC and once with a FC. Differences in canula placement, enhancement and image quality might therefore be biased by overall group differences. Regarding the study design, a completely blind evaluation of catheter placement was not possible as there was an obvious visual difference between the two catheter types. However, the image evaluation was performed blinded. Furthermore, the increased pressure load of the 22-gauge FC in the study resulted in minimal, temporal delay of the contrast agent peak, even though we tried to maximize the potential pressure load through the bolus tracking technique. Another factor in the use of FC is the cost factor, which is many times higher than a conventional catheter. Since this is a retrospective data evaluation, we cannot make any statements about the duration of the application of the respective catheter systems, as this was not recorded. In clinical practice, however, there is no significant time difference with regard to the duration of application. Future evaluations should take the higher cost factor and potential time savings in catheter placement into account. Regarding our sample size, the small number of 37 patients in each group limits the statistical power of the study. Although the antecubital region is the preferred location for catheter placement, the exact positions could not be deduced retrospectively from our reports. As the position of the indwelling venous cannula has an influence on the contrast, thisshould not be omitted as a further limitation of our study. As not the same person was responsible to establish all 78 i.v. catheters, different grades of expertise might have influenced the success rate at the first attempt.

In summary, we were able to show that 22-gauge FCs are equivalent, and partially better in terms of contrast-enhanced image quality and safety compared to a conventional 18-gauge NFC at infusion rates of 5 ml/s in context ofCCTA. The administration of contrast agent with FC may better withstand the recognizable trend of increasingly faster acquisition times with MDCT scanners. With progressively aging patient cohorts, the rise of cardiovascular comorbidities and thus limited peripheral venous conditions, the use of FC might lead not only to faster but also more tolerable examinations due to reduction of catheter sizes. The evaluation of these long-term benefits in clinical praxis could be akey approach for further prospective studies.

## Abbreviations

18-NFC: 18-gauge non-fenestrated catheter
22-FC: 22-gauge fenestrated catheter
CCTA: cardiac CT angiography
CT: Computed tomography
DSCT: dual-source computed tomography
FC: fenestrated catheter
HU: Hounsfield-Units
LA: left atrium
LM: Left main coronary artery
LV: left ventricle
NFC: non-fenestrated catheter
RA: right atrium
RCA: right coronary artery
ROI: regions of interest
RV: right ventricle
SD: standard deviations
WMA: World Medical Association
